# Stem cells as a potential therapy for diabetes mellitus: a call-to-action in Latin America

**DOI:** 10.1186/s13098-019-0415-0

**Published:** 2019-02-18

**Authors:** Mairim Alexandra Solis, Ilais Moreno Velásquez, Ricardo Correa, Lynn L. H. Huang

**Affiliations:** 1 Gorgas Memorial Institute for Health Studies, Panama City, Republic of Panama; 2 Department of Medicine, Warren Alpert School of Medicine, Brown University, Rhode Island, USA; 3 Department of Medicine, University of Arizona College of Medicine, Phoenix, AZ USA; 4 Department of Biotechnology and Bioindustry Sciences, National Cheng Kung University, Tainan, Taiwan; 5 Institute of Clinical Medicine, College of Medicine, National Cheng Kung University, Tainan, Taiwan; 6 Research Center of Excellence in Regenerative Medicine, National Cheng Kung University, Tainan, Taiwan

**Keywords:** Diabetes mellitus, Stem cells, Latin America, Stem cell differentiation, Regenerative medicine

## Abstract

Latin America is a fast-growing region that currently faces unique challenges in the treatment of all forms of diabetes mellitus. The burden of this disease will be even greater in the coming years due, in part, to the large proportion of young adults living in urban areas and engaging in unhealthy lifestyles. Unfortunately, the national health systems in Latin-American countries are unprepared and urgently need to reorganize their health care services to achieve diabetic therapeutic goals. Stem cell research is attracting increasing attention as a promising and fast-growing field in Latin America. As future healthcare systems will include the development of regenerative medicine through stem cell research, Latin America is urged to issue a call-to-action on stem cell research. Increased efforts are required in studies focused on stem cells for the treatment of diabetes. In this review, we aim to inform physicians, researchers, patients and funding sources about the advances in stem cell research for possible future applications in diabetes mellitus. Emerging studies are demonstrating the potential of stem cells for β cell differentiation and pancreatic regeneration. The major economic burden implicated in patients with diabetes complications suggests that stem cell research may relieve diabetic complications. Closer attention should be paid to stem cell research in the future as an alternative treatment for diabetes mellitus.

## Background

Diabetes mellitus (DM), in all its forms, is a metabolic disorder that occurs due to deficient production of insulin by the pancreas. Physiological control of blood glucose levels can be restored in a number of ways: exogenous administration of insulin, medications that stimulate insulin, medications that decrease insulin resistance and/or replace the β cell mass (the producers of insulin) [1]. Pancreatic regeneration of the lost functional β cell mass is an attractive strategy for recovery from the disease. Current approaches utilized for pancreatic replacement of damaged β cells include cadaveric islet transplantation, induction of endogenous regeneration and administration of stem cell-derived β cells [2]. Transplantation of pancreatic islets has proven to be successful for functional replenishment of damaged islets [3, 4]. However, to achieve sustained metabolic control for 1 year, at least 2 million β cells per kg body weight need to be transplanted [5], resulting in a limited availability of healthy islets for this application. The increasing success rate of deriving glucose responsive β-like cells from human stem cells encouraged a new era of β cell replacement therapy, as stem cell therapy could potentially deliver 100–200 million β cells per graft. The current epidemiologic burden of diabetes, globally and especially in Latin America, urges the scientific community to target the key influencers of endogenous β cell regeneration that may be applied to increase the successful rate of differentiated functional β cells, with the hope that in the near future, the load of diabetic patients will be ameliorated through stem cell therapy. Latin America, although at a slow pace, has made important advances in stem cell research. The epidemiology and costs incurred by DM in the region are motivating local stem cell researchers to focus their efforts in developing optimal strategies for obtaining stem cell-derived β cells, which will be the topic of this review.

## Epidemiology and costs of diabetes mellitus: Latin-American perspective

Globally, more than 415 million people were living with DM in 2015 [6]. A 31.1% increase in diabetes-related deaths was reported from 2006 to 2016, leading to 1.43 million deaths in 2016 [7]. In addition, between these years, disability-adjusted life years and years of life lost increased by 24.4 and 25.3%, respectively, reflecting a true global pandemic [8, 9].

According to estimates from the Global Burden of Disease, the burden of DM is greater than expected in Latin America and the Caribbean region [9]. This disease is estimated to affect 10–15% of the adult population in the Caribbean [10] and 9.4% in South and Central-American regions [6]. Recent estimates for DM in Latin America are summarized in Table 1. Furthermore, a strong relationship between socioeconomic deprivation and DM has been consistently reported in several studies, mirroring the social determinants of health in this metabolic disease [9, 11–14]. Recently, a population-based cross sectional study from the Southern Cone of Latin America reported prevalence estimates of DM between 8.4 and 14.3%, with 20% of these cases going undiagnosed [15].

**Table 1:** Relevant recent estimates for diabetes mellitus in the Latin-America and the Caribbean region

Study	International Diabetes Federation [123]	Global Burden of Disease [124]	NCD Risk Factor Collaboration [125]
Region	Central and South America	Latin-American and the Caribbean	Latin-American and the Caribbean
Year	2017	2016	2014
Number or people with undiagnosed diabetes (95% CI)	10.4 million (8.8–12.6 million)		
Age-adjusted comparative prevalence (95% CI)	7.6% (6.3–9.5%)		
Age-standardised DALYs rate per 100 000 (95% CI)		1074.5 (1263.8–83.7)	
Age-standardised incidence rate per 100 000 (95% CI)		306.4 (337.9–279.0)	
Age-standardised diabetes prevalence (men) (95% CI)			8% (6–12%)
Age-standardised diabetes prevalence (women) (95% CI)			9 (6–13%)

*DALYS* disability-adjusted life-years, *CI* confidence intervals

Diabetes is a chronic disease with one of the highest costs to the healthcare system due to its multiple health hazards, high incidence of cardio-metabolic comorbidities, and disabilities that impair individual productivity [16, 17]. Approximately 7% of patients living with DM face costly long-term complications, many of which can be avoided or delayed [18, 19]. Currently, Latin America faces elevated out-of-pocket medical payments [20, 21]. In 2015, The Pan-American Health Organization reported that the average cost of diabetes care per year could range between US $1088 and US $1818, a high amount compared to the gross domestic profit in Latin-American countries [17]. The Prospective Urban and Rural Epidemiological Study revealed that the availability and affordability of essential diabetes medicines are insufficient in low-income and middle-income countries [22]. The current economic burden that diabetes represents prompts scrutiny of the clinical aspects of this pathology for the development of cost-effective treatment strategies.

## Clinical aspects and treatment of diabetes mellitus

Diabetes is an endocrine disorder characterized by hyperglycemia resulting from variable degrees of insulin resistance and/or deficiency [23, 24]. Several forms of diabetes have been described (Table 2). Treatment strategies for diabetes depend on, among other factors, the type of diabetes diagnosed and the severity of the pathology.

**Table 2:** Diabetes classification

Diabetes type	Description
Type 1 diabetes mellitus (T1DM)	β-Cell destruction, usually leading to absolute insulin deficiency
Type 2 diabetes mellitus (T2DM)	Varying degrees of insulin resistance and long-term insulin deficiency
Gestational diabetes mellitus (GDM)	Pregnant women who have never had diabetes mellitus but who experience high blood glucose levels during pregnancy
Maturity-onset diabetes of the young (MODY)	Rare form of diabetes that is distinct from both type 1 and type 2 diabetes and strongly runs in families. It is caused by a mutation in a single gene. If a parent has this gene mutation, any child has a 50% chance of inheriting it
Latent autoimmune diabetes of the adult (LADA)	Disorder in which, despite the presence of islet antibodies at diagnosis of diabetes, the progression of autoimmune β-cell failure is slow
Diseases of exocrine pancreas	Includes pancreatitis, trauma, infection, neoplasia, cystic fibrosis, hemochromatosis, pancreatectomy, and others
Endocrinopathies	Includes acromegaly, Cushing’s syndrome, glucagonoma, hyperthyroidism, somatostatinoma, and others
Drug- or chemical-induced diabetes	Includes some immunotherapy, exogenous steroids, antipsychotics medication, statins, and others
Infections	Congenital rubella and other viruses have been implicated
Uncommon forms of immune-mediated diabetes	Rare cases of diabetes associated with new checkpoint inhibitor therapies
Stiff-man syndrome	An autoimmune disorder of the central nervous system, usually with high titers of glutamic acid decarboxylase [GAD] autoantibodies

Diabetic treatment encompasses an array of lifestyle and pharmaceutical interventions aimed at the prevention of disease progression, hyperglycemia control and mitigation of its micro and macrovascular complications. The treatment options in Latin America include lifestyle modification, hypoglycemic agents and insulin administration [25]. For the past 10 years, several new types of hypoglycemic agents have emerged in the market [26, 27], including metformin, alpha-glucosidase inhibitors, colesevelam, bromocriptine, sulfonylureas thiazolidinediones, dipeptidyl peptidase IV (DPP-4) inhibitors, meglitinide analogs, sodium-glucose cotransporter 2 (SGLT2) inhibitors, and a glucagon-like peptide-1 (GLP-1) receptor agonist. Insulin administration can comprise a simple injection or a more sophisticated insulin pump and closed loop system. However, none of these strategies are able to perfectly control blood glucose, eventually leading to complications. Advances in the development of new therapeutic options, through stem cells, will open the possibility of reversing hyperglycemia and alleviating the many debilitating complications of diabetes. A clear understanding of the pancreatic development and its mechanism of regeneration is critical for the discovery of appropriate treatment strategies for diabetes.

## Insights into pancreatic regeneration: moving beyond conventional diabetes mellitus treatments through stem cell therapy

The pancreas is the major organ that systematically regulates glucose homeostasis. Pancreatic development involves the specific interplay of factors that, among other mechanisms, influence stem cell differentiation into pancreatic progenitor cells and the formation of the fully functional organ. Thus, most stem cell-based differentiation protocols are focused on the generation of mature, single hormone-expressing, glucose-responsive human β cells using information from studies of pancreatic development [28, 29]. Specific signals are involved in the programming of insulin-producing β cells. The transcription factors SRY (sex determining region Y)-box (Sox)17 and homeobox gene HB9 (Hlxb9) are involved in the formation of the endoderm during gastrulation. Following foregut formation, fibroblast growth factor (FGF)-10, retinoic acid, SOX9, and hedgehog signaling pathways induce development of the pancreas. Pancreatic specification and budding then occur through pancreas-specific transcription factor-1a (Ptf-1a), pancreatic and duodenal homeobox 1 (PDX-1), NK6 homeobox 1 (Nkx6.1), neurogenin-3 (Ngn-3), and mafA [30], enabling endocrine formation and consequent stimulation of ISL LIM homeobox 1 (Isl-1), NK2 homeobox 2 (Nkx2.2), neurogenic differentiation factor (NeuroD), paired box gene (Pax)4, and Pax6 signaling that form the islets of Langerhans. The transcription factors Sox17, hepatocyte nuclear factor (HNF)-6, and HNF-3beta (also known as forkhead box A2, Foxa2) are expressed throughout pancreatic development. Finally, FGF-10 and notch signaling-induced stem cell and pancreatic progenitor cell differentiation stimulate neogenesis to create β cells [31, 32].

Discovery of the key factors involved in β cell development has given rise to strategies for obtaining β cells, either by inducing the expression of pancreatic-related transcription factors in distinct types of stem cells or by supplementation of soluble factors during culture. Diverse stem cell models have been used for the successful differentiation of β cell in vitro, including embryonic stem cells, induced pluripotent stem cells, mesenchymal stem cells, and progenitor cells.

### Embryonic stem cells

The best model for pancreatic regeneration studies has been obtained from the use of embryonic stem cells (ESCs). Transgenic expression of PDX-1 and Nkx6.1 was shown to induce the differentiation of ESCs into endocrine cells that are positive for insulin, somatostatin, and glucagon expression [33]. Growth and extracellular matrix factors, including laminin, nicotinamide and insulin, lead to the formation of ESC-derived C-peptide/insulin-positive islet-like cell clusters that release insulin upon glucose stimulation and express Pax4 [34]. Retinoic acid (RA) has important roles in pancreatic development and is widely used to induce pancreatic differentiation of ESCs. When RA is directly added to activin A-induced human ESCs expressing CXCR4, 95% of cells become positive for the pancreatic marker PDX-1 [35]. Animal studies have shown that human ESC-derived glucose-responsive mature β cells encapsulated in alginate and transplanted into a streptozotocin (STZ)-induced diabetic mouse model result in effective glycemic control [36]. However, the ethical implications involved with the use of ESCs have limited their further clinical application. In this respect, induced pluripotent stem cells have been proposed as a suitable alternative cell source with the same pluripotent characteristics as ESCs.

### Induced pluripotent stem cells

Human induced pluripotent stem cells (iPSC) are obtained by reprogramming human somatic cells for the generation of stem cells with pluripotent properties. Human iPSCs have been shown to be an effective cell source for deriving glucose responsive β-like cells [37–40]. Given the complex processes involved in β cell development, it has been difficult to obtain an efficient and replicable β cell differentiation protocol. A potential solution has been proposed to start differentiation from human iPSC-derived PDX-1 and SOX9-expressing pancreatic progenitor cells, which have a prolonged proliferation potential and the ability to produce C-peptide positive β cells [41]. Another efficient differentiation protocol consisted of supplementation of factors involved in epidermal growth factor (EGF), transforming growth factor β (TGF-β), thyroid hormone, and RA signaling, as well as ɤ-secretase inhibition [38], resulting in β cells with the ability to induce Ca^2+^ flux in response to glucose, package insulin into secretory granules, and secrete insulin. It has recently been reported that supplementation of sodium cromoglicate in combination with a previously described protocol causes the induction rate of insulin-positive cells to increase from a mean ± SD of 5.9 ± 1.5% (n = 3) to 16.5 ± 2.1% (n = 3), with increased expression of Ngn-3-positive cells at a mean ± SD of 32.6 ± 4.6% (n = 3) compared to 14.2 ± 3.6% (n = 3) for the non-supplemented control group [42]. Utilization of iPSCs for therapeutic applications involves other major challenges, including recurrent autoimmune attacks in type 1 diabetes, the inherent risk of placing foreign tissue in the body, and potential tumor formation from cells that are not fully differentiated [2]. Fortunately, the use of mesenchymal stem cells has the potential to overcome these barriers.

### Mesenchymal stem cells

Another attractive strategy for obtaining β cells is adult stem cells. Mesenchymal stem cells (MSCs) are considered the most attractive cell source for regenerative medicine. MSCs have been highlighted because of their multi-potentialities, including self-renewal ability, pluripotency, low antigenicity, reduced toxicity, and ease of culture and expansion in vitro to obtain sufficient cells for treatment. These cells are localized in diverse parts of our body, including the bone marrow, adipose tissue, amniotic fluid, umbilical cord blood, and placenta. We have demonstrated that adipose and placenta-derived MSCs (PDMSCs) can be expanded for several passages without losing their self-renewal capacity [43, 44]. The International Society for Cellular Therapy has provided criteria for defining MSCs. As has been previously demonstrated, MSC populations are composed of multipotent cells that are able to adhere to plastic in culture; express the cell surface markers CD105, CD73, and CD90 [45]; lack expression of CD45, CD34, CD14 or CD11b, CD79a, or CD19 and HLADR surface molecules [46]; and have the ability to differentiate into osteoblasts, adipocytes, or chondrocytes [44, 45]. MSCs have also been shown to be able to differentiate into cell types of endodermal and ectodermal lineages [47], including renal tubular cells [48], skin [49], neural cells [50], hepatocytes [51], and insulin-producing cells (IPCs) [52].

MSCs are being extensively investigated in the clinical setting for their immunomodulatory and tissue regenerative properties, as well as their feasibility in the context of islet transplantation [53, 54], demonstrating improved engraftment of pancreatic islets through the suppression of inflammatory damage and immune-mediated rejection. MSC immunomodulatory properties may be mediated through cell–cell interactions and/or secretion of soluble factors [55]. The cell-mediated immune response in MSCs induces T cell activation and leukocyte recruitment to the inflammatory site through CD106. PDMSCs isolated from the chorionic villi have been shown to contain a population of CD106^+^ cells with unique immunoregulatory properties that activate T helper cells and induce tumor necrosis factor (TNF)-α/interleukin (IL)-1b-mediated MSC expansion [56]. PDMSCs secrete soluble factors that mediate immunosuppressive functions through inhibition of lymphocyte proliferation by TGF-β1, hepatocyte growth factor (HGF), prostaglandin E2 (PGE2), and IL-1β and through inhibition of monocyte differentiation into macrophages or dendritic cells through IL-6, IL-10, and macrophage colony stimulating factor [57]. Umbilical cord-derived MSCs (UC-MSCs) cocultured with a hepatoma cell line effectively alleviated palmitic acid and lipopolysaccharide-induced insulin resistance by blocking NLRP3 inflammasome activation and inflammatory agents [58]. When UC-MSCs were infused into type 2 diabetic rats, hyperglycemia was significantly ameliorated, and inflammatory activity was reduced, resulting in improved insulin sensitivity in insulin target tissues. Similarly, in adipose-derived MSCs (AD-MSCs), infusion into diabetic NOD mice reversed hyperglycemia through inducing higher serum insulin, amylin, and glucagon-like peptide 1 levels compared to untreated controls. AD-MSC treatment also reduced CD4^+^ T helper (Th) 1 cells, interferon-γ, and inflammatory cell infiltration, as well as expanded Tregs in a cell contact-dependent manner in vitro and within the pancreas [59]. Administration of bone marrow-MSC-derived extracellular vesicles into mice resulted in the inhibition of antigen-presenting cell activation and suppression of Th1 and Th17 cell development, inhibiting the onset of type 1 diabetes [60]. Other studies have reported the immunomodulatory properties of bone marrow-derived MSCs (BM-MSCs) in islet xenotransplantation, as evidenced by reduced inflammatory markers and increased immune tolerance markers, demonstrating the potential of this strategy in solving transplantation issues of immune-related graft rejection, as described in more detail in the following sections [61]. Interestingly, a recent study in a UC-MSC model demonstrated that MSC-derived IPCs exhibited hypo-immunogenic characteristics in vitro but became immunogenic after transplantation to the host, possibly due to activation from the immune microenvironment [62].

In addition to their immunomodulatory effects, MSCs provide a supportive micro-environmental niche by secreting paracrine factors and depositing extracellular matrix [63]. Evidence has suggested a supportive role for MSCs in the regeneration of endogenous β cells. Studies have demonstrated that BM-MSCs from mice can differentiate in vitro into IPCs and that the differentiated cells express pancreas-specific marker genes [64]. Through genetic manipulation, overexpression of PDX-1 in human BM-MSCs results in differentiation into IPCs [65]. Human BM-MSCs transfected with three genes, PDX-1, Neuro D, and Ngn-3, differentiate into insulin-expressing cells in vitro but lack glucose-responsive insulin expression. However, transplantation of these differentiated cells reduced blood glucose levels in diabetic mice. Interestingly, differentiated IPCs from AD-MSCs intraportally infused into patients exhibited a 30–50% decrease in their insulin requirement, with a 4- to 26-fold increase in serum C-peptide levels [66]. Umbilical cord blood derived embryonic stem cell-like cells that express stage specific antigen 4 (SSEA4) and octamer 4 (Oct4) can differentiate into insulin-producing islet-like cells that express insulin and C-peptide protein [67].

**Fig. 1:**
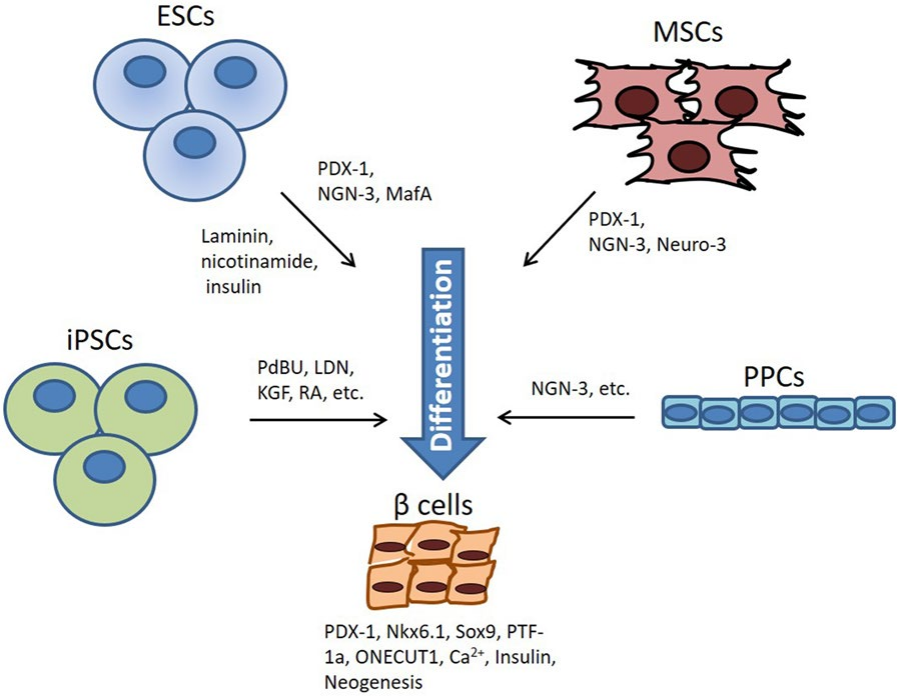
Factors that promote stem cells to induce pancreatic regeneration. Inducible factors that promote ESCs, iPSCs, MSCs, and pancreatic progenitor cells differentiation into β cells, includes among others, PDX-1, NGN-3, Laminin, retinoic acid, among others. *iPSCs* induced pluripotent stem cells, *ESCs* embryonic stem cells, *MSCs* mesenchymal stem cells, *PPCs* pancreatic progenitor cells

Despite the success of the differentiation protocols described in this review, none of these protocols are reproducible for the production of fully functional mature β cells yet. Additional research for the development of more sophisticated differentiation protocols is still required to apply these strategies clinically. Nonetheless, the successful generation of glucose-responsive IPCs through supplementation of crucial factors to the cell-culture medium gives hope for a diabetes treatment derived through stem cell-based cell therapy in the future (Fig. 1). Hence, due to their ease of isolation, immunomodulatory and tissue regenerative properties and the supportive niche they provide by secreting micro-environmental factors and depositing of extracellular matrix, MSCs are suggested to be a suitable stem cell resource for deriving in vitro β cells and for immunomodulation that may prevent graft rejection and autoimmune destruction of β cells.

### Progenitor cells

Identification of progenitor cells in the adult pancreas has received increasing attention due to their pancreatic lineage characteristics that enable them to generate new functional β cells. When pancreatic progenitor cells were induced to differentiate into islets in vitro and transplanted into STZ-induced mice, progenitor cells directly migrated into the injured pancreas, rapidly differentiating into IPCs that decreased glucose levels towards normoglycemia [68]. A recent study demonstrated that progenitor cells expressing Ngn-3, which is expressed at extremely low levels in normal postnatal pancreatic tissues, exists in the ducts of adult mouse pancreas. Ectopic expression of Ngn-3 in pancreatic ductal cells converted them into IPCs, and treatment of human ductal and acinar cells with a combination of epidermal growth factor and gastrin induced neogenesis of islet β cells from the ducts, increasing the functional β cell mass [69]. In other studies, co-transplantation of purified human non-endocrine pancreatic epithelial cells with human fetal pancreatic tissue under the kidney capsule of immuno-deficient mice resulted in their differentiation into endocrine cells. Fetal cells seem to provide factors that support the survival and differentiation of epithelial cells. Stem cell-like cells with the ability to be expanded and form clones ex vivo have also been reported. These cells have the ability to proliferate and form cellular aggregates that display the capacity for endocrine and exocrine differentiation [70]. These results suggest that stem/progenitor cells exist within the pancreas and that these cells might be a source for new islets. However, identification of specific markers is urgently needed for isolation of these cell populations.

### Transplantation of stem cell-derived pancreatic cells

Several types of stem cell-derived pancreatic cells have been proposed for transplantation into diabetic models, including pancreatic progenitors and insulin-secreting cells. As endocrine progenitors differentiate, they migrate cohesively and form bud-like islet precursors. Increasing evidence indicates that proper glucose regulation requires coordination between various islet cell types; therefore, it may be advantageous to produce whole islets in vitro rather than differentiating cells into a specific cell type. A recent study demonstrated obtaining islet precursors from embryonic stem cells, proposing this model to be optimal for obtaining whole islet populations [71].

When conditioned to mature in vivo, transplanted pancreatic progenitor cells produce insulin-secreting cells that prevent or reverse diabetes after transplantation. Transplantation of stem cell-derived pancreatic progenitors on scaffolds that release exendin-4 has been reported to promote the engraftment of stem cell-derived pancreatic progenitors and their maturation toward insulin producing β cells, significantly increasing C-peptide levels and reducing blood glucose in STZ-induced mice [72]. Chronic hyperglycemia and an immunodeficient environment accelerate the maturation of transplanted progenitor cells under the kidney capsule in mice [73, 74]. Pancreatic progenitor cell-to-cell contact before transplantation is crucial for maturation into IPCs in vivo [75]. Nevertheless, in vivo maturation remains a critical issue to be resolved. It is expected that mature endocrine cells generated in vitro would reverse diabetes more rapidly than pancreatic progenitor cells after transplantation. The development of novel techniques is required for in vitro differentiation protocols that may efficiently direct progenitor cells further down the β cell development pathway.

Use of TMP269, a histone inhibitor, in endocrine precursor-like cells derived from Wharton jelly-derived MSCs significantly improved differentiation toward IPCs, as evidenced by the increased expression of PAX4, β- and Δ cells-related genes, and increased secretion of insulin at the end of maturation [76]. Another report describes an improved iPSC-generated endocrine pancreas precursor differentiation protocol that generated populations of greater than 60% insulin-expressing cells that secrete insulin in response to glucose and are capable of reversing diabetes in rodents [77]. In vitro administration of GLP-1 and perGLP-1, the native form of GLP-1, stimulate the differentiation of β cell precursors isolated from mice with diabetes into insulin-producing β cells [78, 79]. Preadipocyte factor 1 (Pref-1) is involved in the proliferation and differentiation of various precursor cells. Overexpression of Pref-1 activates MAPK/AKT signaling, which induces the differentiation of human pancreatic ductal cells into β-like cells with increased insulin synthesis and secretion, and improves glucose homeostasis by accelerating pancreatic ductal and β cell regeneration after injury in a pancreatectomized diabetic animal model [80]. When isolated MSC-like cells from adult mouse pancreas were exposed to an islet differentiation serum-free medium, significant upregulation of the pancreatic markers, Nkx2.2, Nkx6.1, Pdx1, insulin, and somatostatin was observed accompanied by increased insulin secretion over days in culture after glucose exposure [81]. Similarly, incubation of human pancreatic anlage cells with mature β cells resulted in the differentiation of pancreatic anlage into mature β cells. Induced cells acquired the features of mature cells, including increased expression of glucose transporter-2, insulin secretion in response to glucose in vitro, and corrected hyperglycemia in vivo when co-transplanted with vascular cells [82]. Studies have also demonstrated the immunoprophylactic effects of precursors on IPCs. Culture of MSCs in high-glucose media resulted in the generation of precursors to β-like cells with the ability to further differentiate into mature IPCs [12]. Precursor to IPC differentiation seems to more efficiently arrest the autoimmune response in type 1 diabetes when administered before the onset of the disease in NOD mice compared to differentiated IPCs [83].

## Stem cells treatment for diabetic complications

In addition to the generation of IPCs from renewable stem cells, their immunomodulatory, self-renewal, and differentiation properties also suggests MSCs as potentially new therapeutic candidates in the treatment of diabetic-related complications [84]. Retinopathy, critical limb ischemia, and nephropathy are the most common and deleterious diabetic-related complications. Advances in stem cell research have shown the reversal of these complications through stem cell transplant, which will be reviewed below.

### Critical limb ischemia

Peripheral arteriopathy in diabetic patients remains a serious health problem, despite enormous clinical and surgical advances over the last few decades. Stem cell therapy may be a good alternative to major amputation for restoring blood flow and attenuating ischemic disease. Induction of diabetic animals with streptozotocin is a well-defined methodology for the study of peripheral arteriopathy in diabetic models. Our laboratory has established this methodology and demonstrated that a formulated matrix gel induces regenerative neovasculogenesis in the ischemic region (unpublished). AD-MSCs secrete angiogenic and cell survival factors and have been shown to be effective in the treatment of both coronary disease and complications of diabetes in animal and human models [85, 86]. MSC transplantation improves diabetic neuropathy via promoting direct peripheral nerve angiogenesis, neurotrophic effects, and restoration of myelination [87]. A recently reported GMP-compatible protocol described the generation of human ESC-derived endothelial cell products that improve ischemic limb perfusion and local angiogenesis [88]. PDMSC injection into STZ-treated mice demonstrated newly formed capillaries, increased arterioles, and the secretion of proangiogenic factors that promoted ischemic recovery [89].

### Retinopathy

Diabetic retinopathy (DR) is a microvascular complication caused by hyperglycemia in which the retinal blood vessels weaken and rupture due to the chronic degeneration of retinal nerve tissue. Retinal glial cells and pericytes are the earliest-damaged cells with the highest rate of cell death during disease progression, for which cell replacement therapy will be more effective compared to conventional localized treatments. Stem cells have been studied for nerve regeneration in the retina [90]. Recent studies have demonstrated that intravitreal transplantation of neural stem cells originating from human UC-MSCs in DR rats resulted in the long-term preservation of retinal function and significantly delayed the progression of DR for up to 8 weeks with the restoration of vision [91]. It was previously demonstrated that stem cells have protective effects against retinal vasculopathy by preventing capillary loss and retinal capillary dropout in an STZ-induced rodent model of DR [92]. A single intravitreal dose of adipose-derived stem cells along with its secreted paracrine factors was shown to therapeutically improve retina damage through neurovascular repair that led to improved vision [93]. An efficient protocol was reported for generating highly purified human ESC-derived perivascular progenitor cells that demonstrated pericyte marker expression, neural differentiation potential, and improved damaged retinal vasculature after transplantation into a STZ-induced rodent model [94]. In recent clinical studies, autologous BM-MSCs intravenously infused into patients’ eyes exhibited reduced fasting blood glucose, decreased macular thickness reduction, and improved the best corrected visual acuity [95]. Taken together, these studies demonstrate the potential of stem cell-based therapies in the treatment of retinovascular diseases.

### Nephropathy

Diabetic nephropathy is the most common cause of end-stage renal disease and is characterized by alterations of the renal structure and function, including changes in renal tubules, stromal cells, and the incidence of glomerular filtration. MSCs have been shown to relieve diabetic nephropathy through renal tissue repair, modulation of the immune response, and exertion of anti-fibrotic effects [96]. The paracrine effects of MSCs have been shown to increase the regeneration speed of renal tissue during diabetic nephropathy compared to the ability of MSCs to differentiate into renal cells [97]. MSC-derived exosomes improved renal function and repaired renal tissue through autophagic mechanisms in an STZ-induced rat model [98, 99]. In addition, when MSCs are infused in combination with microRNA-124, the results demonstrate the attenuation of renal impairment as well as the inhibition of nephrocyte apoptosis during diabetic nephropathy [100]. Injections of BM-MSCs at the early stages of diabetic nephropathy suppress renal macrophage and cytokine infiltration in diabetic rats, which prevented kidney dysfunction and glomerular defects [101].

## Advances in stem cell research for diabetes treatment in Latin America

Stem cell research in Latin America, although moving at a slow pace, has produced important advances in the establishment of well-equipped laboratories led by specialized local scientists and physicians. Significant advances have been made in stem cell research in Latin America for DM. Potential application of MSCs in the treatment of diabetic neuropathy was demonstrated after observing that preconditioning of human AD-MSCs with increasing concentrations of an iron chelator, deferoxamine, increased the abundance of hypoxia inducible factor 1 alpha, leading to upregulation of pro-angiogenic and neuroprotective factors [102]. On the other hand, studies on diabetic nephropathy from Chile demonstrated that intravenously administered BM-MSCs allowed for pancreatic islet recovery, improved insulin secretion and reversed hyperglycemia in low dose STZ-induced diabetic mice [103]. Further Chilean collaborations with Argentina demonstrated that administration of MSCs to diabetic-induced mice restored pro-regenerative factors, increased the renal proliferation index and anti-inflammatory cytokines levels, and reduced the renal apoptotic index, macrophage infiltration, and oxidative stress damage, resulting in preserved renal function and structure in mice with severe DM. MSC administration completely prevented retinal ganglion cell loss, improving diabetic retinopathy [104]. Donor cells remained in the vitreous cavity and did not differentiate into neural or perivascular-like cells. Nevertheless, they increased the intraocular levels of several potent neurotrophic factors (nerve growth factor, basic fibroblast growth factor and glial cell line-derived neurotrophic factor) and reduced oxidative damage in the retina. MSC administration in diabetic-induced mice showed significant improvement in the functional parameters of kidneys with diabetic nephropathy, with an improved renal proliferation index, decreased renal apoptotic index and restoration of pro-regenerative factors and anti-inflammatory cytokine levels [105, 106]. By contrast, intravenous administration of BM-MSCs neither improved nor impaired diabetic cardiomyopathy in an obesity-induced mouse model [107]. Another research collaboration between Chile and Colombia showed that administration of allogenic BM-MSCs was insufficient for wound healing in diabetic mice, resulting in a delayed therapeutic effect, potentially explained by trophic factors secreted by MSCs being critical for skin regeneration and not the cells per se, suggesting that MSCs may require time to secrete these factors after their administration [108]. In this respect, they showed that MSCs have the capability of restoring the balance between Th1 and Th2 immunological responses along with modification of the pancreatic microenvironment [109].

Pre-clinical animal studies in Brazil demonstrated that betacellulin (BTC), a ligand of the epidermal growth factor receptor, promotes the growth and differentiation of pancreatic β cells and improves glucose metabolism in experimental diabetic rodent models [110]. When MSC-BTC was transplanted into STZ diabetic rats, BTC-transfected cells ameliorated hyperglycemia from over 500 to approximately 200 mg/dL at 35 days post-cell transplantation. Administration of BM-MSCs into diabetic mice reversed hyperglycemia, improved β cell function, and modulated pancreatic cytokine levels [111]. Transplantation of stem cells obtained from murine dental pulp into STZ-induced type 1 diabetic mice improved pancreatic damage and renal function during diabetic neuropathy [112]. AD-MSC treatment reversed hyperglycemia in early-onset diabetes in 78% of diabetic NOD mice, and this effect was associated with higher serum insulin, amylin, and glucagon-like peptide 1 levels compared to untreated controls. In addition, AD-MSC treatment ameliorated autoimmune diabetes pathogenesis in diabetic NOD mice by attenuating the Th1 immune response concomitant with the expansion/proliferation of Tregs, thereby contributing to the maintenance of functional β cells [59]. Co-transplantation of rat-derived BM-MSCs with pancreatic islets into mice resulted in reduced expression of inflammatory markers, such as TNFs, chemoattractant protein 1, and IL-1b, along with increased immune tolerance markers, IL-4, IL-10, and forkhead box P3, demonstrating the immunomodulatory actions of BM-MSC [61].

Brazil launched clinical trials testing the ability of autologous BM-MSCs to reverse diabetes, stroke and heart conditions. Seventeen clinical trials in progress are utilizing AD-MSCs, especially in cardiology, orthopedics, diabetes and neurology. High-dose immunosuppression and hematopoietic stem cell (HSCs) transplant performed with acceptable toxicity in a small number of patients with newly diagnosed type 1 DM has shown increased β cell function in all but 1 of 18 patients with prolonged insulin independence in the majority of patients [113]. Administration of autologous HSCs into 21 type 1 diabetic patients resulted in all patients becoming insulin independent for a period of 43 months with induced immunoregulation that consisted of lower CD3^+^CD4^+^ T cell numbers and consistent CD3^+^CD8^+^ T-cell levels, resulting in a CD4/CD8 ratio inversion. Memory cytotoxic T cells comprised the majority of T cells detected, and B cells returned to baseline levels 2–3 months post-transplantation. Baseline islet-specific T-cell autoreactivity persisted after transplantation, but regulatory T cell counts increased. Patients with lower frequencies of autoreactive islet-specific T cells remained insulin-free longer and presented greater C-peptide levels than those with lower frequencies of these cells [114].

Mexico reported that administration of autologous HSCs demonstrated increased C-peptide synthesis and insulin independence in most type 1 DM patients, with a decrease in HbA1c levels [115]. In addition, a protocol to isolate PDX-1-expressing IPCs from MSCs was developed by inducing expression of Nestin in MSCs, followed by a short incubation of 24 h in low glucose medium, and finally, a longer incubation of 168 h in high glucose medium [116]. Another study reported full ulcer recovery of a patient with chronic foot ulcer after MSC transplantation [117].

Findings from Argentina demonstrated that the combined therapy of intra-pancreatic AD-MSC infusion and hyperbaric oxygen improved metabolic control and reduced insulin requirements in patients with type 2 DM [118]. In general, Argentina’s governmental support is strong and a driving force in stem cell research. Currently, 0.65% of the country’s GDP is invested in science and technology, which is a model that should be followed in additional Latin-American countries.

The latest strategy for the restoration of the β cell mass is through the generation and transplantation of stem cell-derived β cells [119], indicating that related research will be beneficial to Latin America (Table 3). Our shared vision is that these countries will maintain their effort in promoting innovative excellent research, establishing or improving regulations to the highest international level, increasing regional and international cooperation and identifying country- or region-specific opportunities to collaborate worldwide without diminishing identity or sovereignty.

**Table 3:** Stem cell research in diabetes in some Latin-American countries

Country	Stem cell type	Diabetes application outcome	References
Mexico	MSC	In vitro-β cell differentiation	[116]
		Patient full recovery of chronic foot ulcer	[117]
	HSC	Patient Insulin Independence	[115]
Chile	MSC	In vitro improved diabetic neuropathy	[102]
		In vivo improved diabetic retinopathy	[104]
		In vivo improved diabetic nephropathy	[105, 106]
		In vivo improved diabetic cardiomyopathy	[107]
		In vivo improved pancreatic environment	[109]
Brazil	MSC	Pancreatic immunomodulation	[61, 114]
		Reverse hyperglycemia, improve β cell function and/or pancreatic immunomodulation	[59, 111, 120]
		In vivo β cell differentiation and function	[110]
	Dental pulp stem cells	In vivo β cell renewal, prevent renal damage	[112]
	HSC	Patient improved β cell function and insulin independence	[121]
Argentina	HSC	Patient improved pancreatic environment	[122]
	ADSC	Improve metabolic control and reduce insulin requirements	[118]

*MSC* mesenchymal stem cell, *HSC* hematopoietic stem cell, *ADSC* adipose-derived stem cell

## Conclusion

Diabetes is a global health and economic burden in which the disability-adjusted life years and years of life lost represent a problematic issue in Latin America. A unique set of challenges exist for DM treatment, as the diabetic prevalence has increased over the years. Latin America urgently needs to reorganize its health care services to optimize diabetes therapeutic goals. Recent breakthroughs in deriving glucose responsive β-like cells from human stem cells has provided encouragement for β cell replacement therapy. More evidence is demonstrating the potential for embryonic stem cells, adult stem cells, and progenitor cells to produce β cells with the ability to produce insulin, reduce glucose levels in animal models, and to some extent, reverse diabetes symptoms through pancreatic regeneration. Stem cell research groups in Latin America have focused their efforts and provided important contributions to the DM field. Success in the generation of glucose-responsive IPCs and MSC-induced immunomodulation gives hope for the development of improved diabetic treatments through stem cell-based cell therapy in the near future.

## Abbreviations

DM: diabetes mellitus
MODY: maturity-onset diabetes of the young
LADA: latent autoimmune diabetes of the adult
DPP-4: dipeptidyl peptidase IV
SGLT2: sodium-glucose cotransporter 2
GLP-1: glucagon-like peptide-1
NPH: neutral protomaine hagedorn
Sox: SRY (sex determining region Y)-box
Hlxb9: homeobox gene HB9
FGF-10: fibroblast growth factor 10
Ptf-1a: pancreas-specific transcription factor-1a
PDX-1: pancreatic and duodenal homeobox 1
Nkx6.1: NK6 homeobox 1
Ngn-3: neurogenin-3
Isl-1: ISL LIM homeobox 1
Nkx2.2: NK2 homeobox 2
NeuroD: neurogenic differentiation factor
Pax: paired box gene
HNF: hepatocyte nuclear factor
Foxa2: forkhead box A2
ESC: embryonic stem cells
RA: retinoic acid
STZ: streptozotocin
EGF: epidermal growth factor
TGF-β: transforming growth factor beta
iPSC: induced pluripotent stem cells
MSC: mesenchymal stem cell
PDMSCs: placenta-derived MSCs
IPCs: insulin-producing cells
TNF: tumor necrosis factor
IL: interleukin
HGF: hepatocyte growth factor
PGE2: prostaglandin E2
UC-MSC: umbilical cord-derived MSCs
Th: T helper
BTC: betacellulin
BMSC: bone marrow-derived MSC
SSEA4: stage specific antigen 4
Oct4: octamer 4
DR: diabetic retinopathy
NSC: neural stem cell
HSC: hematopoietic stem cell
PPC: pancreatic progenitor cell
